# Interfaceless Exchange Bias in CoFe_2_O_4_ Nanocrystals

**DOI:** 10.1021/acs.nanolett.2c04268

**Published:** 2023-02-27

**Authors:** Beatriz Rivas-Murias, Martín Testa-Anta, Alexander S. Skorikov, Miguel Comesaña-Hermo, Sara Bals, Verónica Salgueiriño

**Affiliations:** † CACTI, Universidade de Vigo, 36310 Vigo, Spain; ‡ Institut de Ciència de Materials de Barcelona (ICMAB-CSIC), Campus de la UAB, 08193 Bellaterra, Spain; § Electron Microscopy for Materials Research (EMAT), University of Antwerp, Groenenborgerlaan 171, 2020 Antwerp, Belgium; ∥ Université Paris Cité, CNRS, ITODYS, Paris, F-75013 Paris, France; ⊥ Departamento de Física Aplicada, Universidade de Vigo, 36310 Vigo, Spain; # CINBIO, Universidade de Vigo, 36310 Vigo, Spain

**Keywords:** Exchange bias, spinel ferrite nanocrystals, pinned and unpinned uncompensated moments, Raman spectroscopy

## Abstract

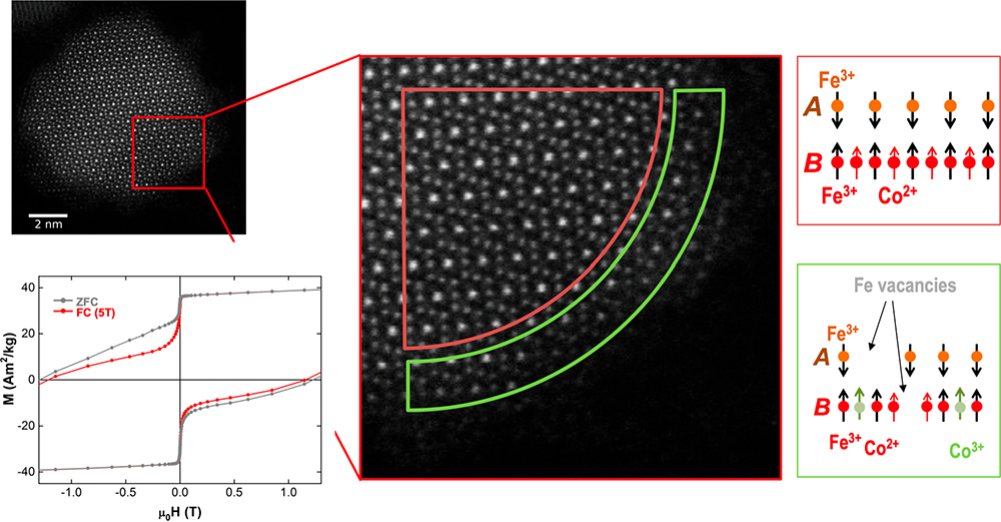

Oxidized cobalt ferrite nanocrystals with a modified distribution of the magnetic cations in their spinel structure give place to an unusual exchange-coupled system with a double reversal of the magnetization, exchange bias, and increased coercivity, but without the presence of a clear physical interface that delimits two well-differentiated magnetic phases. More specifically, the partial oxidation of cobalt cations and the formation of Fe vacancies at the surface region entail the formation of a cobalt-rich mixed ferrite spinel, which is strongly pinned by the ferrimagnetic background from the cobalt ferrite lattice. This particular configuration of exchange-biased magnetic behavior, involving two different magnetic phases but without the occurrence of a crystallographically coherent interface, revolutionizes the established concept of exchange bias phenomenology.

The exchange bias (EB) effect, also referred to as unidirectional or exchange anisotropy, describes a magnetic coupling observed in core–shell nanocrystals (NCs) or thin films, generally between an antiferromagnet (AFM) and a ferro- or ferrimagnet (FM and FiM, respectively) separated by a physical interface.^1^ The EB effect with FM/FM, FiM/FiM, AFM/AFM, or FM/spin glass exchange interactions has also been reported,^2−6^ as well as more exotic systems stemming from interfacial spin configurations (that is, noncollinear or frustrated interface spins)^6−8^ or even in magnetic NCs holding antiphase boundaries due to their strained crystalline structure.^9,10^ Such coupling produces a horizontal shift in the hysteresis loop after cooling under an applied magnetic field and is often accompanied by an increase in its coercive field (*H*_C_), endorsing these systems with a huge relevance in many technological applications related to permanent magnets^11^ or magnetic recording media.^12,13^

Given that EB is by definition an interfacial phenomenon dependent on a physical boundary between two well-differentiated magnetic components,^1,14^ fine-tuning of the dimensions, nature, and overall quality of such interface is needed in order to control the magnetic coupling.^15,16^ In this context, thin interfacial layers with FM or AFM properties generated at film–substrate interfaces, driven by a structural^17^ or magnetic reconstruction,^18^ as well as spin disorder,^19^ can add new degrees of freedom for engineering across heterointerfaces.

The origin of EB is known to lie on pinned uncompensated interfacial spins,^20^ but a crucial influence of the inner (bulk) pinned uncompensated spins from the AFM component was recently demonstrated.^21^ In fact, the interfacial spin distribution can be modified by the bulk AFM magnetic landscape, for instance, via nonmagnetic impurities or crystallographic defects, both of them conducive to AFM order dilution and the consequent AFM domain formation.^21,22^ These phenomena have been investigated in AFM materials, but the underlying physical mechanism can be considered for their FiM counterparts.

Among the FiM candidates for the development of EB systems, combinations of different spinel ferrites stand out, given their potential in spintronics.^23,24^ These spinel-type oxides are prone to disorder and exchange processes on the cation sublattices with the normal and inverse spinel as the two limiting cases under an ordered sublattice occupation. The disorder, if controlled, can perturb the ideal local coordination, for instance, by inducing charge imbalances and ion vacancies, all of this having a huge impact on the heterostructure behavior. Consequently, the electrical and magnetic properties of these ferrites and, in general, of the transition metal oxide heterostructures,^25−27^ can be modified when tailoring the interfacial and lattice characteristics through ionic motion.^28^ Along these lines, a systematic tuning of the atomic distribution at the tetrahedral and octahedral sites of these spinel ferrites has opened new pathways for generating emergent phenomena in heterostructures due to ion migration.^29−31^ Nevertheless, some control in the ion migration is required to avoid the otherwise deleterious effects degrading the EB.^32^

Still, despite the important advances made in understanding the EB effect,^1,21^ its analysis in single-phase objects lacking a core–shell or a layered structure, that is, lacking a physical interface between two magnetic phases, has been reported scarcely.^11,18^ Herein, we present a confined chemical treatment at the surface of single-crystallite CoFe_2_O_4_ NCs by which a change in the spinel crystalline structure is not appreciated, but an ionic rearrangement in the subsurface and surface regions of the spherical NCs is induced. This situation offers a unique exchange coupling interaction within the same NC, without establishing a physical or coherent crystallographic interface. Yet, the magnetization reversal of the modified NCs is observed to occur in two steps and to come along with an increase in coercivity and an EB shift, suggesting the existence of a strong exchange interaction between two magnetic components. The chemical changes registered, associated with the cation rearrangement in the spinel structure, help understand the magnetism displayed and underline the possibilities of this new chemical route for the engineering of EB-related functionalities for final device applications.

CoFe_2_O_4_ NCs with a spherical shape and narrow size distribution (10.6×/1.3 nm average diameter (95.5%), log-normal fit) were synthesized by seed-mediated growth (experimental details and Figure S1 in the Supporting Information (SI)). Figure 1a includes a TEM image of the NCs, and Figure 1b shows its powder XRD pattern at room temperature, which is indexed to a cubic spinel structure (*Fd3̅m* symmetry group) and allows the disposal of secondary phases. The cell parameter obtained from the Le Bail analysis is 0.8394 nm (see Table S1.1 in the SI), in good agreement with bulk cobalt ferrite (JCPDS card 22-1086).^33−35^ Elementary analysis using ICP-OES indicates an average Co_0.95_Fe_2.05_O_4_ stoichiometry (from now on termed CoFe_2_O_4_). A fraction of the same batch of these CoFe_2_O_4_ NCs was subsequently immersed and confined in a basic aqueous medium using a water-in-oil (W/O) reverse microemulsion, that is, stabilizing them by a nonionic surfactant (Igepal CA-520) in water droplets in a hydrophobic continuous phase.^36^ Besides the surfactant, these water droplets of very small volume exposed the NCs to a high pH, promoting an oxidation process at their surface.^36,37^ This sample is hereafter labeled as CoFe_2_O_4_@Ox.

**Figure 1:**
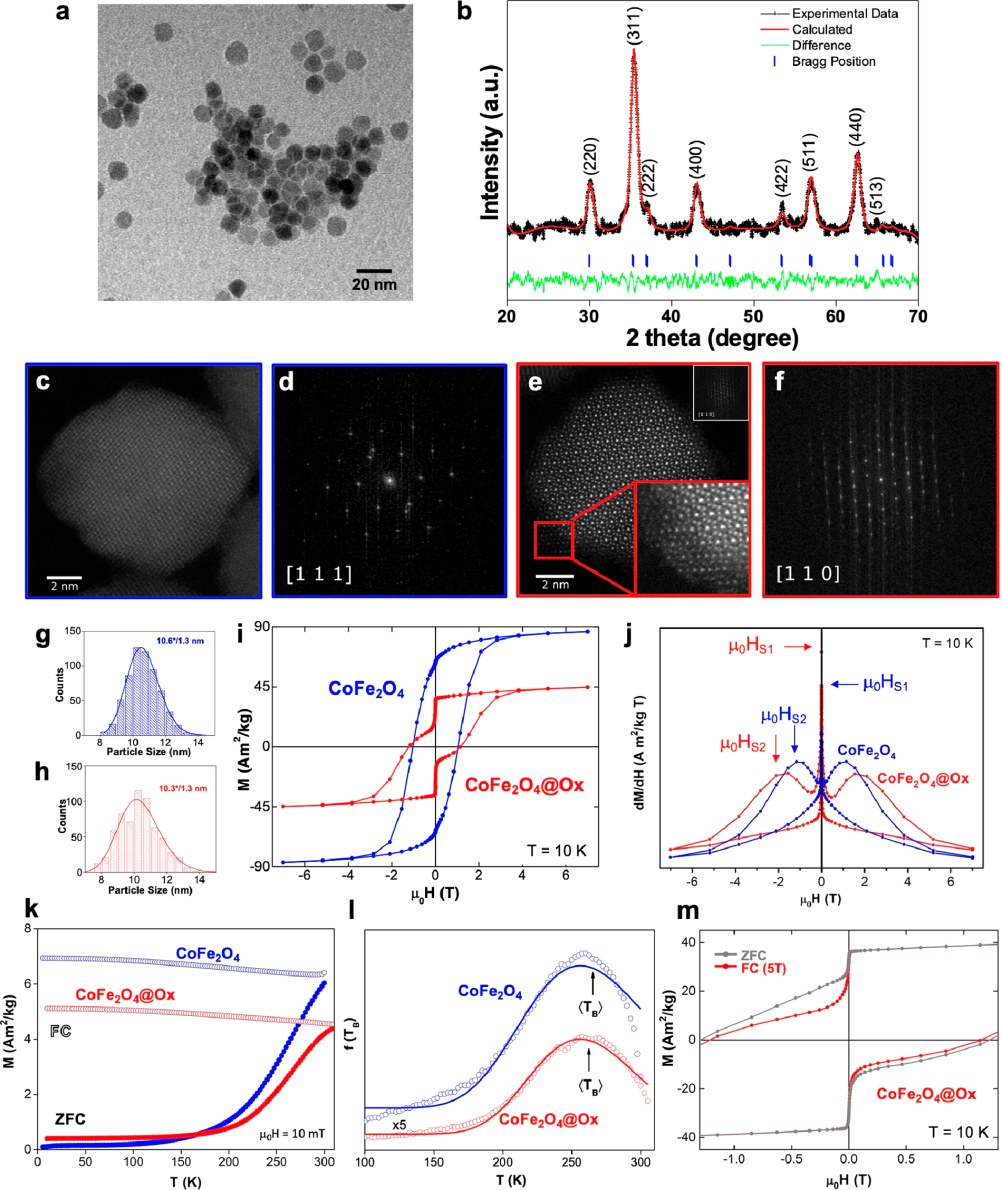
TEM image with general particle overview and X-ray diffraction pattern with Le Bail refinement of the CoFe_2_O_4_ NCs (a, b). HAADF-STEM images of representative CoFe_2_O_4_ (c) and CoFe_2_O_4_@Ox (e) NCs and their respective FFT images (d, f). Inset in e: zoomed-in image of the defect-free crystalline structure. Size histograms (fitted to log-normal functions) of CoFe_2_O_4_ (g) and CoFe_2_O_4_@Ox (h) NCs. Hysteresis loops measured at 10 K of CoFe_2_O_4_ (blue) and CoFe_2_O_4_@Ox (red) samples (i) and comparison of their derivatives with μ_0_H_S1_ and μ_0_H_S2_ magnetic fields at which two events of magnetization switching occur (j). ZFC and FC curves measured at 10 mT (k) and distribution of energy barriers *f*(*T*_B_) calculated from the ZFC-FC curves and derivatives (l) of CoFe_2_O_4_ (blue dots) and CoFe_2_O_4_@Ox (red dots) samples. Hysteresis loops measured at 10 K of the CoFe_2_O_4_@Ox sample after ZFC (red) and FC at 5 T (gray) (m).

To shed light on the effect these conditions exert on the CoFe_2_O_4_ spinel structure, a high-angle annular dark-field scanning transmission electron microscopy (HAADF-STEM) study of the as-synthesized (Figure 1c and d and Figure S2a and c, CoFe_2_O_4_ sample) and the chemically treated (Figure 1e and f and Figure S2b and d, CoFe_2_O_4_@Ox sample) NCs was performed. These images show no clear differences in the crystalline structure of these representative NCs, lacking in both cases the core–shell structure expected from a superficial oxidation. Indeed, the spinel crystalline lattice highlighted at these high resolution images does not have defects, dislocations, or twin boundaries up to the surface. The higher resolution image included in Figure 1e, obtained along the [110] zone axis, permits us to appreciate distinct contrast associated with the positions of the atomic columns, offering an enlarged view of the defect-free spinel structure in the whole nanocrystal from the CoFe_2_O_4_@Ox sample. Interestingly, we can appreciate neither Moiré fringes nor grain or antiphase boundaries.^38^ Moreover, the size histogram of the samples (fitted to log-normal function) does not show apparent modifications either (Figures 1g and h), and the analysis of atomic column positions in a 2D projection of a CoFe_2_O_4_@Ox NC shows the absence of systematic strain fields (Figure S3), thus excluding the presence of interfacial strain between two crystalline phases.

Yet, the magnetic behavior of the two CoFe_2_O_4_ and CoFe_2_O_4_@Ox samples points to an important change in the configuration of the magnetic cations in the spinel structure. Figure 1i includes the comparison of the magnetic properties displayed by the as-synthesized CoFe_2_O_4_ sample (blue curve) and by the CoFe_2_O_4_@Ox sample (red curve) at 10 K. The value of maximum magnetization registered for the CoFe_2_O_4_ sample is ∼87 Am^2^/kg when applying the maximum field (7 T), close to that of the bulk cobalt ferrite saturation magnetization (*M*_S(bulk)_ ∼ 90 Am^2^/kg)^39^ and similar to other values reported for NCs.^40,41^ This is in agreement with the very good crystallinity of the NCs observed by HAADF-STEM and the stoichiometry registered. Anyhow, different effects related to a canted surface spin structure,^42^ a gradient in the magnetic cations ratio moving outward from the core to the surface,^43−45^ or a cationic disorder in the crystalline structure at the surface can explain the slight difference. While this value of *M*_S_ when applying the maximum 7 T field drops to ∼45 Am^2^/kg for the CoFe_2_O_4_@Ox sample, the coercive field value increases (μ_0_*H*_C_ = 1.06 T and μ_0_*H*_C_ = 1.26 T for CoFe_2_O_4_ and CoFe_2_O_4_@Ox samples, respectively). In both cases, these high values of μ_0_*H*_C_ are related to the high magnetostriction of CoFe_2_O_4_, due to the strong spin–orbit coupling from the Co^2+^ ions in the crystalline lattice.^43,46^ In addition, the shape of the hysteresis loop has evolved after the chemical microemulsion-based treatment, with two reversals of magnetization (one of them much larger), seen as two inflection points (μ_0_*H*_S1_ and μ_0_H_S2_ magnetic fields) around 5 and 10 mT and 1.14 and 1.55 T when comparing the derivatives (dM/dH), shown in Figure 1j. The significantly larger reversal contribution at low field in the CoFe_2_O_4_@Ox sample clearly hints that a cationic modification took place. Similar contributions at low field were reported in samples of CoFe_2_O_4_ NCs synthesized by a coprecipitation method under alkaline conditions,^34,47,48^ where phase segregation (due to Co_3_O_4_ and/or Fe_2_O_3_) or a phase with a reduced crystallinity was formed, but not detected in our samples in STEM. Figure 1k displays the temperature dependent magnetization curves, measured under ZFC (zero-field-cooled) and FC (field-cooled) conditions and recorded applying a field of 10 mT. Whereas the mass magnetization value of the CoFe_2_O_4_@Ox has decreased notably in comparison to that of the initial sample (in line with the hysteresis loops), the shapes of the ZFC and FC curves are very similar. Based on these ZFC-FC curves, it is possible to estimate the energy barrier distribution (in terms of the blocking temperature, *T*_B_: *f*(*T*_B_) ∝ (1/*T*) [*d*(*M*_ZFC_ – *M*_FC_)/*dT*],^49−51^ fitted to a log-normal function in agreement with the size distribution; see Figure 1g,h,l). The average *T*_B_ values obtained are very similar, 265 and 262 K for CoFe_2_O_4_ and CoFe_2_O_4_@Ox samples, respectively, and comparable to other values in the literature.^40,52−54^ This match in the blocking temperature reflects the very similar magnetically coherent volumes of FiM material in both samples,^55^ despite the fact that there are two reversals of magnetization. Such a finding is in line with the absence of an interface or any other crystalline defect in the crystalline structure and the absence of byproducts (smaller nanoparticles and/or low anisotropy magnetic phases).

In order to further analyze the switching behavior in the CoFe_2_O_4_@Ox sample in terms of exchange-coupling properties, we measured the hysteresis loops under ZFC and FC conditions applying an external magnetic field of 5 T (Figure 1m). Though there is a decrease in coercivity, the FC hysteresis loop shows a negative field shift (μ_0_*H*_E_= −56.9 mT) associated with an EB effect. Such coupling, usually attributed to a FM/AFM interaction, is strong enough to produce a unidirectional anisotropy that causes the observed shift. The reduced coercivity registered for the CoFe_2_O_4_@Ox sample under FC conditions (compared to that of the ZFC loop) also unveils a reduction of the effective magnetic anisotropy, likely induced by the large cooling field. Similar trends have been reported in previous studies and have been ascribed to AFM order frustration.^56−58^ In our case, this observation can be understood considering the presence of pinned uncompensated spins, which align with the sufficiently large cooling field employed and induce frustration of the FiM exchange coupling. The presence of this hypothesized larger number of pinned uncompensated moments is supported experimentally by the large drop in saturation magnetization but increased coercivity. Overall, these results point to a crucial influence of the synthetic conditions on the magnetic behavior of the CoFe_2_O_4_@Ox sample with respect to the as-synthesized one.

Aiming to corroborate the idea of the confined chemical effect in the micelles as the origin of the change in the magnetic behavior observed for the CoFe_2_O_4_@Ox sample, we performed an additional mapping of Fe and Co distributions and investigated their electronic configuration using electron energy loss spectroscopy (EELS). Figure 2a shows elemental mapping for CoFe_2_O_4_ and CoFe_2_O_4_@Ox samples, which indicates a similar increasing concentration of Co toward the surface of the nanocrystal in both cases. Although we cannot completely exclude an effect of electron beam irradiation, the results of different experimental techniques corroborate that CoFe_2_O_4_ or MnFe_2_O_4_ NCs synthesized via thermal decomposition typically have an increased content of Co or Mn at their surface owing to the different decomposition temperatures of the metallic precursors.^43−45,59^ In this regard, the hysteresis loops of the initial sample show a very small reversal of magnetization at low field which can stem from the cobalt patches observed at the surface and present in both native and oxidized samples. Anyway, the very large value of coercivity registered at low temperature can only be associated with the Co_*x*_Fe_3–*x*_O_4_ stoichiometry, even with increasing values of *x* as moving outward. Additionally, EELS spectra of L edges for Fe and Co in CoFe_2_O_4_ and CoFe_2_O_4_@Ox samples (Figure 2b) reveal that (a) the L edge of Co in CoFe_2_O_4_@Ox is shifted by *ca*. + 1.0 eV in comparison to CoFe_2_O_4_, indicating an increase in the oxidation state of Co, resulting from the chemical treatment^60,61^ and (b) the ratio between the Fe L_3_ and Co L_3_ edges decrease from 2.95 in the CoFe_2_O_4_ sample to 2.59 in the CoFe_2_O_4_@Ox sample, indicating an average decrease in the iron content. These experimental results point to a partial oxidation of cobalt cations and the formation of Fe vacancies in the spinel structure at the subsurface region, providing an explanation for the presence of pinned uncompensated moments associated with the changes in the magnetic behavior of the CoFe_2_O_4_@Ox sample and, particularly, to the EB effect.

**Figure 2:**
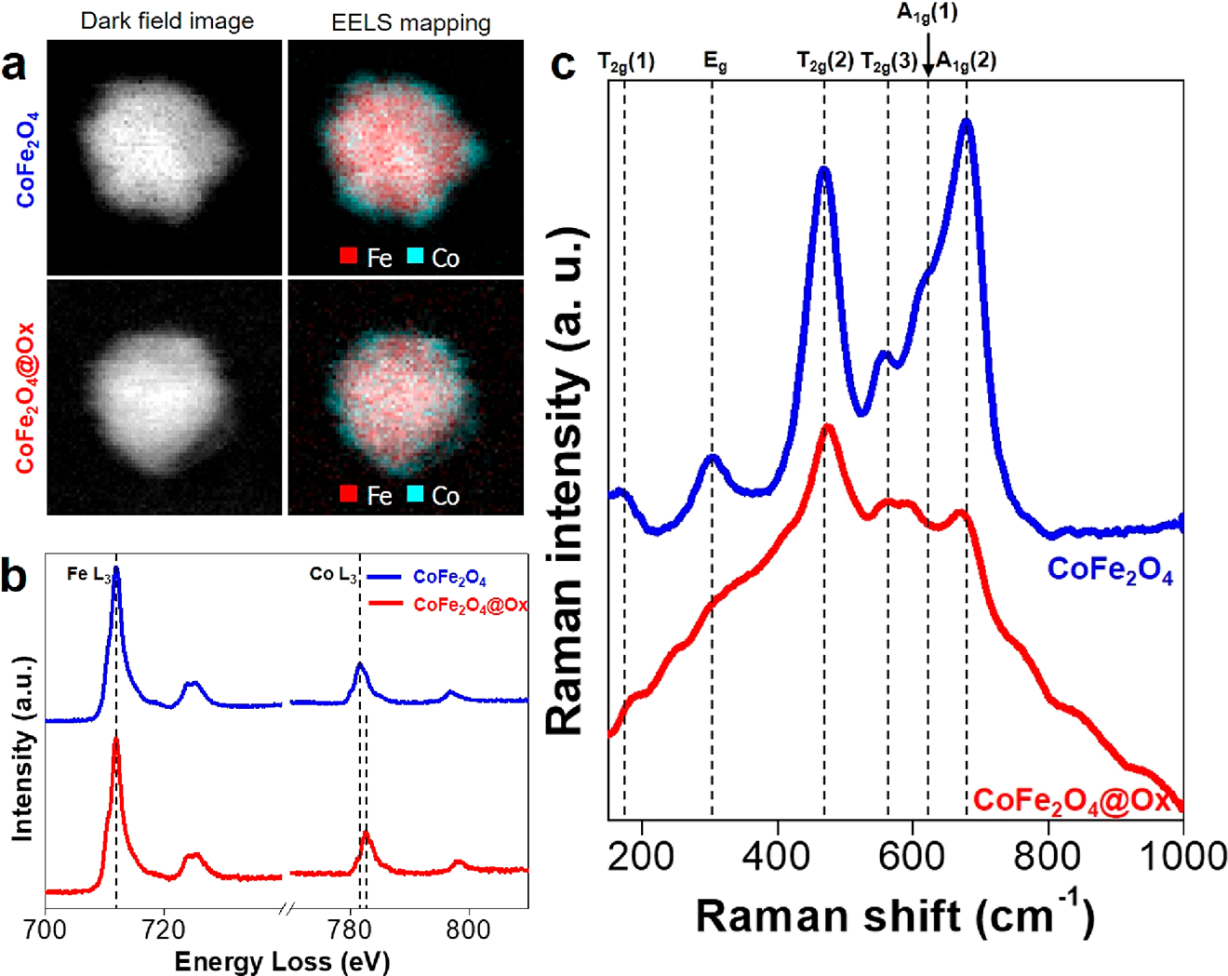
(a) Mapping of Fe and Co distributions in CoFe_2_O_4_ and CoFe_2_O_4_@Ox NCs based on EELS. (b) Comparison of EELS spectra for L edges of Fe and Co between those of CoFe_2_O_4_ and CoFe_2_O_4_@Ox samples. The dashed lines in the Co L_3_ edge point out the shift of the edge position after oxidation. (c) Stokes-shifted Raman spectra registered using a 785 nm excitation wavelength from the CoFe_2_O_4_ (blue curve) and CoFe_2_O_4_@Ox (red curve) samples.

To further support this relationship, we performed a Raman analysis of the as-synthesized CoFe_2_O_4_ and CoFe_2_O_4_@Ox samples in order to investigate the chemical and structural origin of the peculiar magnetic features observed.^62^ This technique can register six Raman active modes from the spinel structure, namely, 2*A*_1*g*_, *E*_*g*_, and 3*T*_2*g*_, characteristic of spinels with two different types of cations occupying the octahedral or tetrahedral sites, such as CoFe_2_O_4_ (see Figure S4 in the SI).^63^ In general, the ferrite *A*_1*g*_ modes appear above 600 cm^–1^ and are usually assigned to the motion of oxygen in the tetrahedral AO_4_ group along the ⟨111⟩ direction, involving a symmetric stretching of the oxygen atoms with respect to the metal ion at the tetrahedral void (T),^64^ as well as the deformation of the three octahedral sites (O) nearest to each oxygen.^65^Figure 2c includes the Raman spectra registered. In the as-synthesized sample (blue spectrum), the expected modes for the spinel lattice are observed,^66^ with the most intense peak at 679 cm^–1^ (*A*_1*g*_(2)) and a small shoulder at ∼620 cm^–1^ (*A*_1*g*_(1)), which stem from the presence of Fe^3+^ and Co^2+^ ions at the tetrahedral sites, respectively.^65,67^ Note that the *A*_1*g*_(1)) mode intensity is much lower than that of *A*_1*g*_(2), meaning that the primary contribution to the AO_4_ vibrations originates from the Fe^3+^ ions. There is less consensus regarding the origin of the other low-frequency modes (*E*_*g*_ and *T*_2*g*_), typically assigned to the tetrahedral unit in the Fe_3_O_4_ material^64,68^ or to the octahedral unit when considering mixed spinel ferrites such as CoFe_2_O_4_ or ZnFe_2_O_4_.^69,70^ In the latter case, the *E*_*g*_ vibrational mode has been assigned to the symmetric bending of oxygen with respect to Fe in the octahedral BO_6_ void^64^ and is usually absent in nanocrystals.^71^ The fact that this mode can be ascertained in our spectrum underlines once again the optimal crystallinity of the CoFe_2_O_4_ NCs. On the other hand, the *T*_2*g*_(2) mode has been reported to account solely for the Co^2+^ ions occupying the octahedral sites.^72^ Hence, the higher intensity of the *T*_2*g*_(2) and the *A*_1*g*_(2) modes compared with the *A*_1*g*_*(*1) mode confirms the predominant inverse spinel configuration anticipated for the cobalt ferrite.

The characteristic features of the spinel crystalline structure are still present in the CoFe_2_O_4_@Ox spectrum (red curve). However, the relative intensities of the *A*_1*g*_ and *T*_2*g*_ vibration modes have notably changed, the *T*_2*g*_(2) mode (at ∼470 cm^–1^) being the most prominent feature in the Raman spectrum. Interestingly, the vibrational modes *T*_2*g*_(3) and *A*_1*g*_(1) begin to merge into one broad band owing to the pronounced red-shift of the *A*_1*g*_(1) mode, now located at ∼600 cm^–1^ (see also Figure S4). This shift is usually associated with structural distortions and/or the presence of a different set of cations at the tetrahedral/octahedral sites. Taking into account the absence of strain fields (Figure S3) and the *ca*. +1.0 eV shift observed in the Co L edge from the CoFe_2_O_4_@Ox sample (compared to CoFe_2_O_4_; Figure 2b), the red-shift of the *A*_1*g*_(1) mode is consistent with the presence of Co^3+^ cations within the spinel structure, in addition to Fe^3+^ and Co^2+^.^67^ The resultant charge compensation of the crystalline structure may proceed via Fe vacancies^73^ or a partial Fe^3+^/Fe^2+^ reduction. While the presence of Fe^3+^ vacancies is supported by the decrease in the Fe/Co L_3_ ratio registered, which indicates a reduced iron content in the CoFe_2_O_4_@Ox sample with respect to the pristine sample, the partial Fe^3+^/Fe^2+^ reduction seems less probable given the absence of observable changes in the L edge of the Fe spectrum in the EELS analysis. The fact that we have not registered the vacancies in the HAADF-STEM analysis suggests that their content must be rather small. Along these lines, the decrease in the intensity of the *A*_1*g*_(2) mode can be explained by this chemical and local modification promoted by the Fe^3+^ vacancies created. The oxidation of some of the Co^2+^ ions to Co^3+^ and the changes associated with the Fe^3+^ ions raise the question whether a nonstoichiometric Co^II^Co^III^Fe^III^□O_4_ spinel is formed at the subsurface region, but since the HAADF-STEM analysis reveals no evidence of two crystallographic phases at the core and the surface shell, the as-formed mixed ferrite spinel must be highly disordered in terms of the metallic cation distribution. This disorder is also hinted at by the presence of additional features in the Raman spectrum of the CoFe_2_O_4_@Ox sample, displaying new modes of low intensity at 420 and 760 cm^–1^, for instance.

An additional experiment registering the evolution of the Raman spectra as a function of the incident laser power was also performed for the two CoFe_2_O_4_ and CoFe_2_O_4_@Ox systems (Figure S5). The Raman spectrum of the as-synthesized sample evolves into the same signature observed for CoFe_2_O_4_@Ox when treated under a laser power of 5.82 mW (Figure S5a), which can be associated with a partial oxidation of Co^2+^ cations reported above.^74^ Furthermore, the very similar spectra recorded at 0.42 mW, after subjecting both samples to the highest laser power (21 mW; Figure S5a and b), exhibit a notable increase in the *T*_2*g*_(2) mode intensity compared to the *A*_1*g*_(2) mode, in agreement with a reduced iron content due to Fe^3+^ vacancy formation. The presence of these vacancies can be understood as a preliminary step prior to the transformation toward maghemite (γ-Fe_2_O_3_) and is also corroborated by the blue-shift of the *A*_1*g*_(2) mode to ∼690 cm^–1^ (note that the *A*_1*g*_ mode characteristic from maghemite occurs at ∼700 cm^–1^).

Conclusively, to explain the coupling mechanism and the local magnetic configuration given the chemical changes registered by EELS and Raman spectroscopy and given the fact that there is no crystallographically coherent interface, we take the coercivity of the initial CoFe_2_O_4_ NCs as a reference (μ_0_*H*_C_ = 1.06 T). With this large value taken into account, the fraction of the CoFe_2_O_4_ phase at the outer shell of the as-synthesized NCs switches readily (μ_0_*H*_S1_ = 5 mT; Figure 1j). However, for the CoFe_2_O_4_@Ox sample, while the magnetic phase at the subsurface region now switches with a value of μ_0_*H*_S1_ = 10 mT, the CoFe_2_O_4_ phase at the core follows an even larger switching field (μ_0_*H*_S2_ = 1.55 T). This can be explained by considering the presence of unpinned uncompensated moments in the cation disordered subsurface region of the CoFe_2_O_4_@Ox sample, which couple to the external field and rotate along with the FiM CoFe_2_O_4_ core, resulting in a coercivity enhancement.^21,22^ On the other hand, the rather strong negative μ_0_*H*_E_ field (−56.9 mT) indicates the presence of pinned uncompensated moments that strongly couple to the FiM lattice but do not rotate even at the maximum field (7 T). The presence of these pinned and unpinned magnetic moments can be understood as the outcome of competing interactions within the parental spinel structure, where the Co^2+^ oxidation has the Fe–O–Fe and Co–O–Fe superexchange interactions disrupted, leading to a highly frustrated subsurface region. This increased magnetic frustration, boosted by the assumed cation disorder, is reflected not only in the drop in the value of magnetization down to 45 Am^2^/kg (which can be explained by the presence of low-spin Co^3+^ on octahedral sites^75^) but also in the low-anisotropy component detected during the reversal of the CoFe_2_O_4_@Ox sample. This fact hints that, besides inducing magnetic disorder, some short-range correlated spin disorder occurs. This situation, particularly in terms of the effects stemming from the presence of Co^3+^ and the iron vacancies, inducing a charge reorganization, with local modifications of the valence charge states and possible creation of defect gap states, can explain the changes not only in the magnetization but also in the electronic, ionic, and tunnel conductivities.^76^ Such effects require a more in-depth investigation that falls out of the scope of this study. Alternative scenarios in the attained cation distribution at the subsurface, such as a change from an inverse to a normal spinel configuration,^77,78^ would lead to a magnetization enhancement and can be therefore discarded.

In summary, whereas the HAADF-STEM images show NCs with a uniform crystalline structure up to the surface and without the presence of defects or strain, both EELS and Raman spectroscopy, jointly with the magnetic properties, point to the presence of a pseudo core–shell structure with no physical interface. Such unusual characteristics render the system particularly fascinating, pointing to interfaceless exchange coupling between two different distributions of magnetic cations within the parent spinel structure.

## Supporting Information Available

Experimental methods, supplementary Le Bail refinement data, HAADF-STEM data, strain analysis, supplementary magnetic data, and supplementary Raman spectroscopy analysis (PDF)
