# Supplementary material for: Interfaceless Exchange Bias in CoFe2O4 Nanocrystals
Source: Nano Lett. 2023 Feb 27;23(5):1688–95. doi: 10.1021/acs.nanolett.2c04268 (PMC10848284; doi:10.1021/acs.nanolett.2c04268)
Supplement: Supplementary file 1 — nl2c04268_si_001.pdf [file nl2c04268_si_001.pdf]

**Supporting Information**

# Interfaceless Exchange Bias in $CoFe_2O_4$ Nanocrystals

*Beatriz Rivas-Murias,[1] Martín Testa-Anta,[2] Alexander S. Skorikov,[3] Miguel Comesaña-Hermo,[4] Sara Bals,[3] Verónica Salgueiriño[5,6]**

[1]CACTI, Universidade de Vigo, 36310 Vigo (Spain)

[2]Institut de Ciència de Materials de Barcelona (ICMAB-CSIC), Campus de la UAB, 08193 Bellaterra (Spain)

[3]Electron Microscopy for Materials Research (EMAT), University of Antwerp, Groenenborgerlaan 171, 2020 Antwerp (Belgium)

[4]Université Paris Cité, CNRS, ITODYS, Paris F-75013 Paris (France)

[5]Departamento de Física Aplicada, Universidade de Vigo, 36310 Vigo (Spain)

[6]CINBIO, Universidade de Vigo, 36310 Vigo (Spain)

## Experimental

**Materials:** Iron(III) acetylacetonate (Fluka, 97%). Cobalt(II) acetylacetonate (97%), 1,2-dodecanediol (90%), oleylamine (70%), oleic acid (90%), benzyl ether (98%), poly (5)oxyethylene-4-nonyphenylether (Igepal® CA-520), cyclohexane (99%), tetraethyl orthosilicate (TEOS, 98%), ammonium hydroxide (28-30%), hexane (95%) and absolute ethanol were purchased from Sigma-Aldrich and used as received.

**Synthesis of $CoFe_2O_4$ Seeds:** $CoFe_2O_4$ nanocrystals were synthesized by thermal decomposition, using a seed mediated growth process. The method employed is based on a previously reported synthesis procedure,[1,2] though with slight modifications. 20 mL of benzyl ether were added to a mixture of 1 mmol of $Co(acac)_2$, 2 mmol of $Fe(acac)_3$, 10 mmol of 1,2-dodecanediol, 6 mmol of oleic acid and 6 mmol of oleylamine in a 100 mL three-neck round-bottom flask. After degassing this solution with nitrogen at 70°C for 30 minutes with magnetic stirring, the mixture was firstly heated to 200°C (heating rate of 8°C/min) and kept for 2 hours. Subsequently this solution was heated to reflux up to 250°C at the rate of 5°C/min$^{-1}$, was kept at this temperature for one hour, and was finally left to cool down to room temperature. During all the process the solution was magnetically stirred at 300-400 rpm.

**Growth of $CoFe_2O_4$ Nanocrystals:** Similar conditions were used to obtain ~10 nm $CoFe_2O_4$ nanocrystals. In particular, 84 mg of $CoFe_2O_4$ seeds previously synthesized were dispersed in 4 mL of hexane and added to a solution of 20 mL of benzyl ether, 1 mmol of $Co(acac)_2$, 2 mmol of $Fe(acac)_3$, 10 mmol of 1,2-dodecanediol, 2 mmol of oleic acid and 2 mmol of oleylamine in a 100 mL three-neck round-bottom flask, previously degassed in nitrogen for 30 minutes at 70ºC. The hexane of the mixture was firstly evaporated at 100°C during 30 min to remove the hexane and the solution was then heated up to 200°C at 8°C/min$^{-1}$for 1 hour. Subsequently, this solution was heated up to reflux to 250°C, at 5°C/min$^{-1}$ for 30 min. Then again, the reaction was left to cool down to room temperature and the magnetic stirring was maintaining at 300-400 rpm during all the process.

In order to wash the samples, in both cases absolute ethanol was added to the brown-black solutions and then centrifuged at 9000 rpm for 20 minutes. The black collected samples of seeds or nanocrystals of cobalt ferrite were washed three times (9000 rpm/10 min) with a mixture of hexane and ethanol. Finally, the nanocrystals were dried and stocked in powder.

**Synthesis of $CoFe_2O_4$@Ox Nanocrystals:** $CoFe_2O_4$ nanocrystals were immersed in a basic pH using a reverse microemulsion system based on the procedure described by Koole *et al.* for semiconductor quantum dots.[3] For the microemulsion-based process oxidation, 1.3 mL of Igepal® CA-520 was dispersed in 10 mL of cyclohexane and mechanically stirred for 15-18 min (850 rpm).

Subsequently, 50 μL of a 42 mM solution of $CoFe_2O_4$ nanocrystals was added. After 15 minutes of stirring between each step, 500 μL of ammonium hydroxide were added into the solution. After this last step, the mixture was stirred for 3 hours.

**Characterization:** The X-ray diffraction (XRD) pattern was collected using a Panalytical X'Pert Pro diffractometer (Cu $K_\alpha$ radiation, Bragg-Brentano θ-2θ geometry) in the 2θ angular range of 20-80° using a continuous scan mode (step=0.026°, 4s per step). The XRD pattern were analyzed using Rietica software. Inductively Coupled Plasma- Optical Emission Spectroscopy (ICP-OES) analysis was performed on Perkin Elmer Optima 4300 DV spectrometer to quantify the amount of cobalt and iron into $CoFe_2O_4$ nanocrystals. Transmission electron microscopy (TEM) images were performed on a JEOL JEM1010 instrument operating at an acceleration voltage of 100 kV. Size histograms were obtained by manual counting over a minimum of 200 particles in each sample considering diameter. The corresponding size distributions were fitted to a log-normal function, expressing the final results in terms of the geometric mean and standard deviation as x */2σ (i.e. for a 95.5% interval of confidence).[4] High-angle annular dark-field scanning TEM (HAADF-STEM) imaging was performed using an aberration-corrected Thermo Fisher Titan electron microscope operated at accelerating voltage of 300 keV. For electron energy loss spectroscopy

(EELS), accelerating voltage of 120 kV was used, and the electron beam was monochromated, resulting in about 100 meV full width at half maximum for the zero-loss peak. We used 50 mA electron beam current and about 10 minutes of total exposure per EELS map. The acquired EELS maps were analyzed using Gatan Microscopy Suite 3.51 and HyperSpy 1.7.2 software.

Samples for TEM and HAADF-STEM analysis were prepared by dropping a diluted suspension of the nanocrystals onto an ultrathin carbon coated copper grid. Raman spectra were collected with a Renishaw in Via Reflex Raman Microscope. Experiments were conducted at room temperature using a 785 nm laser excitation wavelength. The spectra were compared with reference Raman standards for different oxides ($CoFe_2O_4$, $Co_3O_4$, CoO, FeO, $Fe_3O_4$ and $Fe_2O_3$).[5-7] The laser beam was focused on the sample by a 50x objective, with a numerical aperture (NA) value of 0.75. The laser power on the sample surface used was 1% or 5%, in order to avoid any possible modifications on the surface sample. Magnetic measurements were performed using the vibrating sample magnetometer (VSM) option in a Physical Property Measurement System (PPMS) from Quantum Design. The magnetization dependence with temperature in zero-field-cooling (ZFC) and field-cooling (FC) conditions was performed at 100 Oe in the 10-320 K range. Hysteresis loops under

ZFC and FC conditions were measured at 10 and 300 K up to an external field of 7T. The magnetic measurements were performed using dried powdered samples.

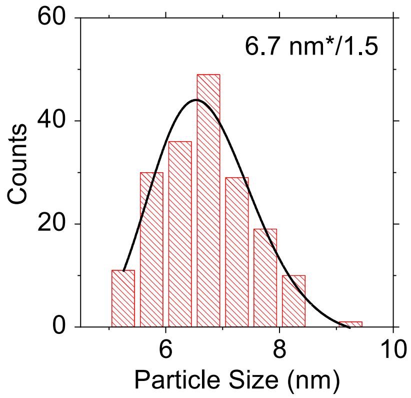


**Figure S1.** Size histogram of $CoFe_2O_4$ seeds used for the subsequent growth of the $CoFe_2O_4$ NCs.

| | | | |
|---|---|---|---|
| **Cell parameter:** *a* = 0.8394 nm | | | |
| **Background:** Cheby 12 terms | | | |
| **Peak Shape:** Pseudo-Voigt (How. Asym) | | | |
| **Instrumental Peak Shape** | | | |
| **U** | -2.10 | **Asy1** | -0.249 |
| **V** | 2.70 | **Gam 0** | 0.858 |
| **W** | 0.073 | | |
| **Goodness of fit parameters (R indices)** | | | |
| $R_{exp}$ | 8.71 | $R_B$ | 0.04 |

| | | | |
|---|---|---|---|
| **$R_{wp}$** | 10.98 | $\chi^2$ | 1.59 |
| **$R_p$** | 8.26 | | |

**Table S1.1.** Parameters refined, fit results and R- or goodness of the fit parameters in the Le Bail refinement performed on the XRD pattern of the initial the $CoFe_2O_4$ NCs, included in Figure 2b.

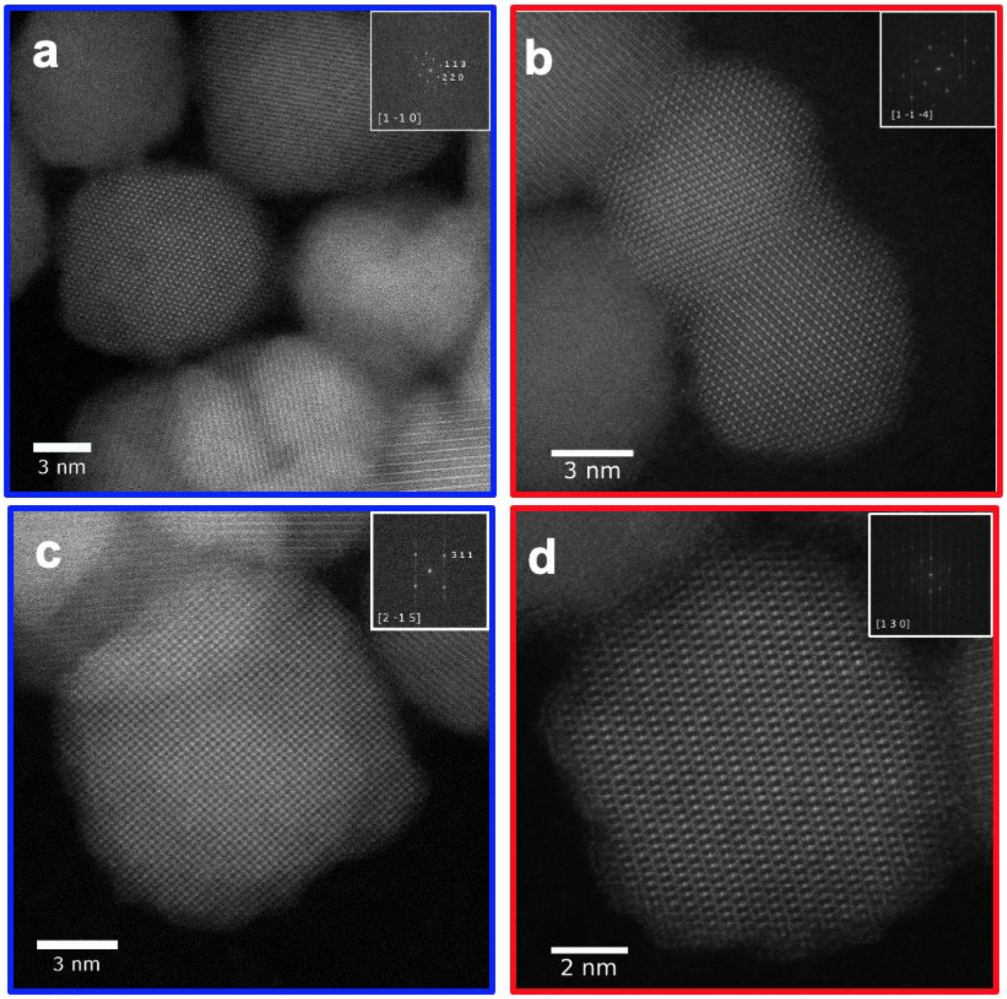


**Figure S2.** HAADF-STEM images of representative $CoFe_2O_4$ (a, c) and $CoFe_2O_4$@Ox (b, d) NCs and their respective FFT images (insets), reflecting the defect-free crystalline structure.

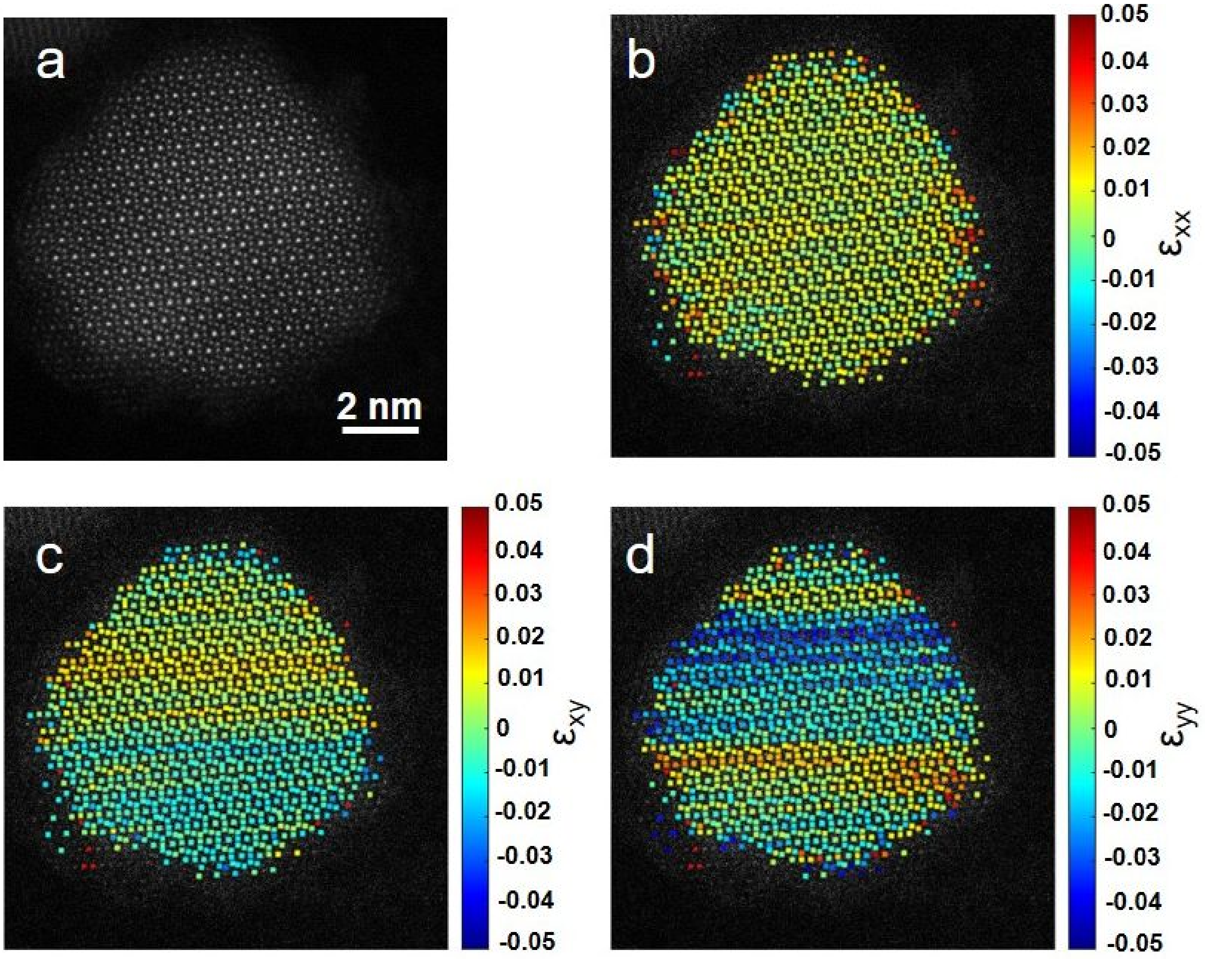


**Figure S3.** 2D strain analysis of the $CoFe_2O_4$@Ox sample based on the positions of atomic columns determined using StatSTEM 3.1.1 software. (a) HAADF-STEM image and (b-d) strain mappings of a single NP. The slight strain in the vertical direction (slow scanning direction) is most likely related to the drift of the sample.

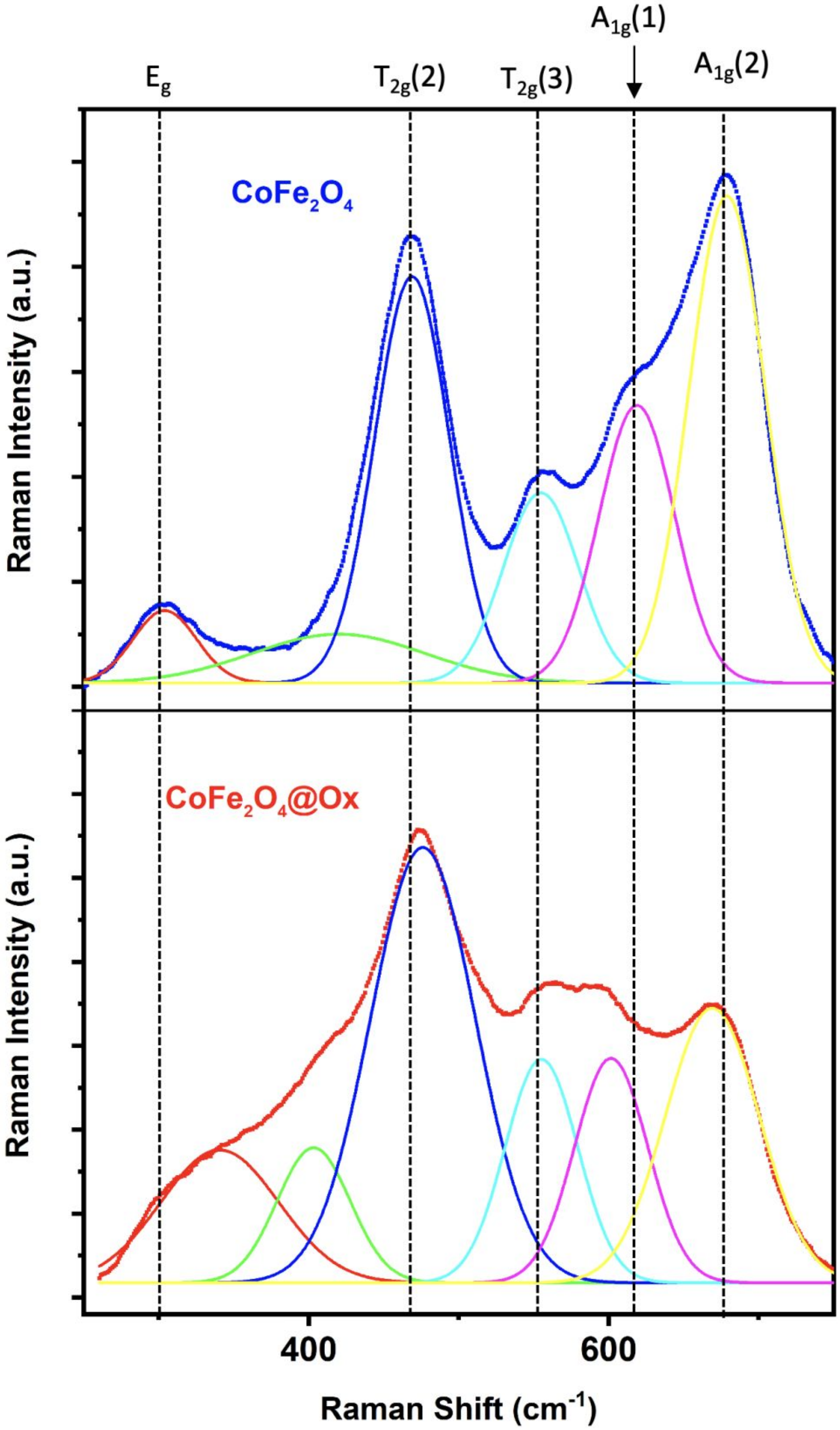


**Figure S4.** Stokes-shifted Raman spectra registered using a 785 nm excitation wavelength for $CoFe_2O_4$ (top) and $CoFe_2O_4$@Ox (bottom) samples, respectively, with the Raman active modes fitted to Gaussian curves, for a closer inspection.

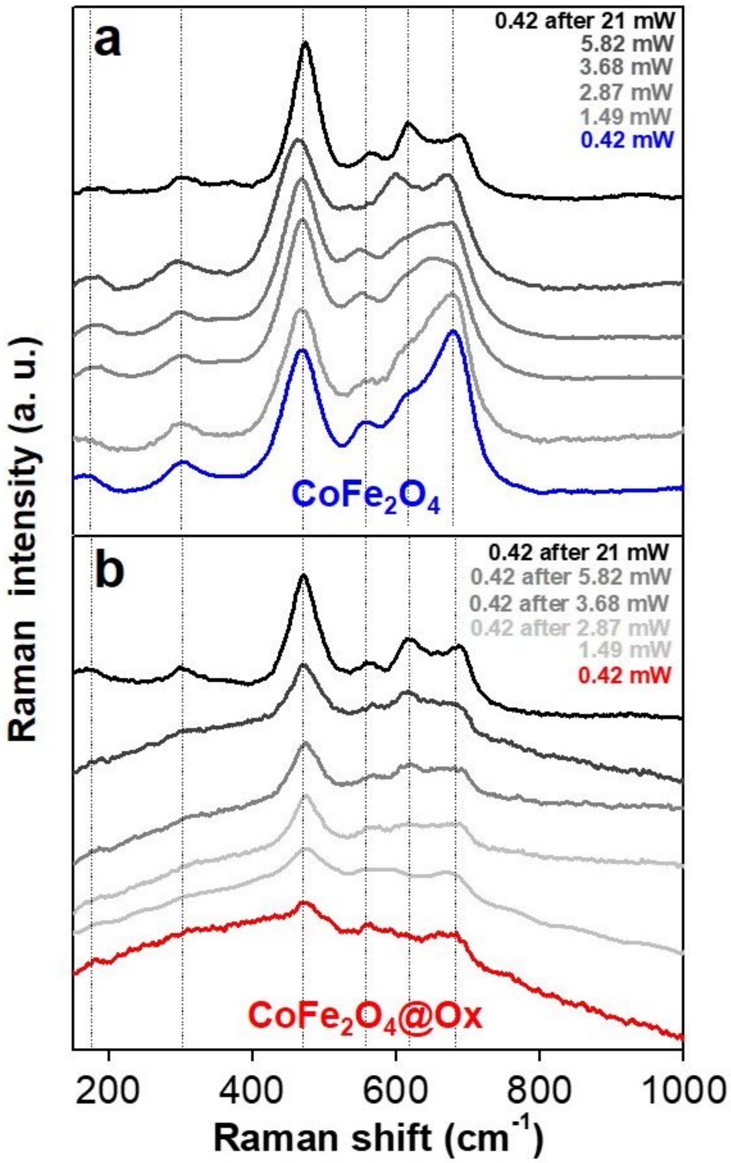


**Figure S5.** (a, b) Stokes-shifted Raman spectra evolution registered using a 785 nm excitation wavelength as a function of the laser power for $CoFe_2O_4$ and $CoFe_2O_4$@Ox samples, respectively. This kind of analysis on dark samples such as the present ones imply heating effects because of a local increase in temperature, that promote different processes such as phase transitions or even the decomposition of the sample.[5] They can also induce important modifications of the cationic distribution at the tetrahedral/octahedral sites of the spinel structure. Interestingly, under these

conditions, the as-synthesized sample evolves into the same signature observed for $CoFe_2O_4$@Ox sample when treated under a laser power of 5.82 mW (Figure S3a), offering the same crystalline transition as the chemical treatment. Furthermore, the spectra recorded at 0.42 mW after subjecting the samples to the highest laser power (21 mW) is the same for both, highlighting the reproducibility of this crystalline crossover.

## References


(1) Sun, S.; Zeng, H. Size-Controlled Synthesis of Magnetite Nanoparticles. *J. Am. Chem. Soc.* **2002**, *124*, 8204–8205.

(2) Sun, S.; Robinson, D. B.; Raoux, S.; Rice, P. M.; Wang, S. X.; Li, G. Monodisperse MFe2O4 (M = Fe, Co, Mn) Nanoparticles. *J. Am. Chem. Soc.* **2004**, *126*, 273–279.

(3) Koole, R.; Van Schooneveld, M. M.; Hilhorst, J.; De Donegal, C. M.; 'T Hart, D. C.; Van Blaaderen, A.; Vanmaekelbergh, D.; Meijerink, A. On the Incorporation Mechanism of Hydrophobic Quantum Dots in Silica Spheres by a Reverse Microemulsion Method. *Chem. Mater.* **2008**, *20* (7), 2503–2512.

(4) Limpert, E.; Stahel, W. A.; Abbt, M. Log-Normal Distributions across the Sciences: Key and Clues. *BioScience* **2001**, *51*, 341-352.

(5) Rivas-Murias, B.; Salgueiriño, V. Thermodynamic CoO-$Co_3O_4$ Crossover using Raman Spectroscopy in Magnetic Octahedron-shaped Nanocrystals. *J. Raman Spectroscopy* **2017**, *48*, 837-841.

(6) Testa-Anta, M.; Ramos-Docampo, M. A.; Comesaña-Hermo, M.; Rivas-Murias, B.; Salgueiriño, V. Raman Spectroscopy to unravel the Magnetic Properties of Iron Oxide Nanocrystals for Bio-related Applications. *Nanoscale Adv.* **2019**, *1*, 2086-2103.

(7) Chandramohan, P.; Srinivasan, M. P.; Velmurugan, S.; Narasimhan, S. V. Cation Distribution and Particle Size Effect on Raman Spectrum of $CoFe_2O_4$. *J. Solid State Chem.* **2011**, *184*, 89-96.
